# Late esophageal wall injury after mesh repair for large esophageal hiatal hernia: a case report

**DOI:** 10.1186/s40792-017-0401-4

**Published:** 2017-12-15

**Authors:** Kentaro Yatabe, Soji Ozawa, Eisuke Ito, Junya Oguma, Akihito Kazuno, Miho Nitta, Yamato Ninomiya

**Affiliations:** Department of Gastroenterological Surgery, Tokai University School of Medicine, 143 Shimokasuya, Isehara, Kanagawa 259-1193 Japan

**Keywords:** Hiatal hernia, Mesh erosion, Migration

## Abstract

**Background:**

Plication of an esophageal hiatus during surgery for esophageal hiatal hernia is a common practice; however, a mesh may be used if the hiatus is markedly enlarged. Recently, various late complications occurring as a result of mesh-induced esophageal and/or gastric wall injuries have been reported.

**Case presentation:**

A 71-year-old woman presented at a neighborhood clinic in November 2010 with chief complaints of respiratory distress on exertion and heartburn. She was diagnosed as having a large esophageal hiatal hernia and was treated at our hospital using a laparoscopic Toupet fundoplication with mesh repair of the esophageal hiatus. Two years and 1 month after the operation, the patient complained of a bowel obstruction. An upper gastrointestinal endoscopy revealed that part of the mesh had extruded into the esophageal lumen, resulting in ulceration and stricture of the esophageal wall. Endoscopic balloon dilatation failed to improve the esophageal stricture. In July 2012, the patient underwent a lower esophagectomy with proximal gastrectomy and was discharged on the 25th hospital day.

**Conclusions:**

We experienced a rare case requiring surgical treatment for a mesh-induced esophageal wall injury after surgery for a giant esophageal hiatal hernia. The selection of a soft, durable mash and its firm securement at a position distant from the gastrointestinal wall may be important to avoid late esophageal wall injury.

## Background

Plication of an esophageal hiatus during surgery for esophageal hiatal hernia is a common practice; however, in patients with a markedly large hiatus, a mesh may be used to reinforce the weakened diaphragmatic crura and to close a hiatus that cannot be plicated. Unfortunately, various late complications arising from mesh-induced injuries to the esophageal and/or gastric wall have begun to be reported [1]. Here, we report a patient who required surgery because of an esophageal injury caused by a mesh used in a previous surgery for the repair of a large esophageal hiatal hernia.

## Case presentation

A 71-year-old woman visited a neighborhood clinic in November 2010 with chief complaints of respiratory distress on exertion and heartburn. She was referred to us for further medical workup under a suspected diagnosis of esophageal hiatal hernia. Her past history and family history were unremarkable. At the first visit, a physical examination yielded no positive findings, and hematologic/blood biochemical tests also showed no abnormalities. However, a plain chest X-ray revealed a stomach gas bubble that had obliterated the cardiac silhouette within the mediastinum. An abdominal computed tomography (CT) and upper gastrointestinal series revealed an intrathoracic herniation of the stomach*,* with slight torsion of the corpus ventriculi. The patient was thus diagnosed as having a type IV esophageal hiatal hernia and underwent a laparoscopic Toupet fundoplication in February 2011.

**Fig. 1:**
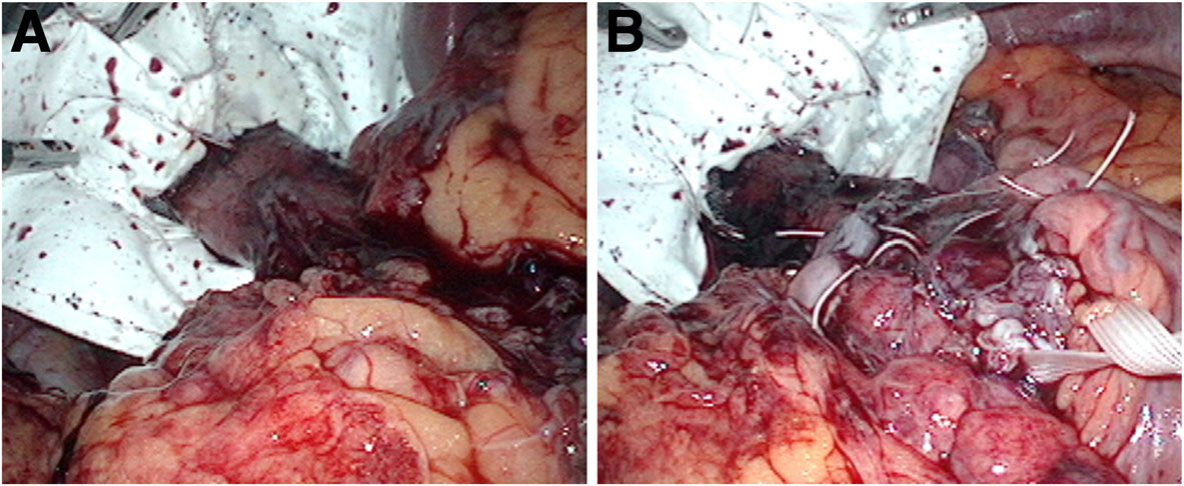
**a** An operative field after the mesh repair. **b** An operative field after Toupet procedure

The intraoperative findings revealed a markedly enlarged esophageal hiatal orifice, resulting in the intrusion of the stomach and the greater omentum into the esophageal hiatus. The stomach and greater omentum that had been drawn into the mediastinum through the enlarged esophageal hiatal orifice were restored to their intraperitoneal positions. The esophageal hiatus was plicated dorsally and then ventrally; however, durable closure of the hiatus seemed difficult by this method alone, and mesh reinforcement was subsequently undertaken. A Bard Composite Mesh (PTFP/ePTFP) was cut from the margin to the center and shaped so as to create a keyhole around the abdominal esophagus, with appropriate clearance. To reinforce the hiatus, the mesh was fixed in place using sutures at a distance of about 5 mm from the abdominal esophagus (Fig.1 (A)). Fundoplication was then performed using the Toupet procedure (Fig.1 (B)). The patient had an uneventful postoperative course and was discharged on the 7th hospital day.

**Fig. 2:**
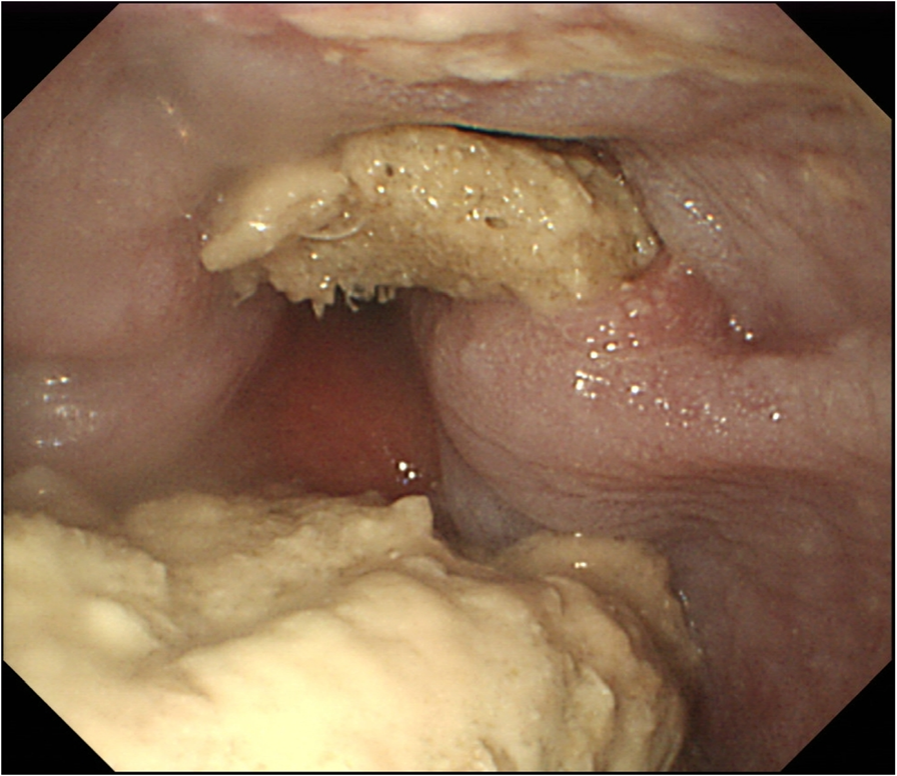
An endoscopic examination revealed exposed mesh

**Fig. 3:**
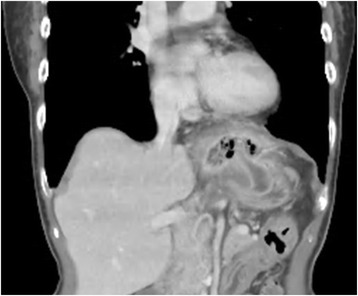
A CT examination shows the extrusion of the mesh into the peri-esophageal region and the esophageal lumen

**Fig. 4:**
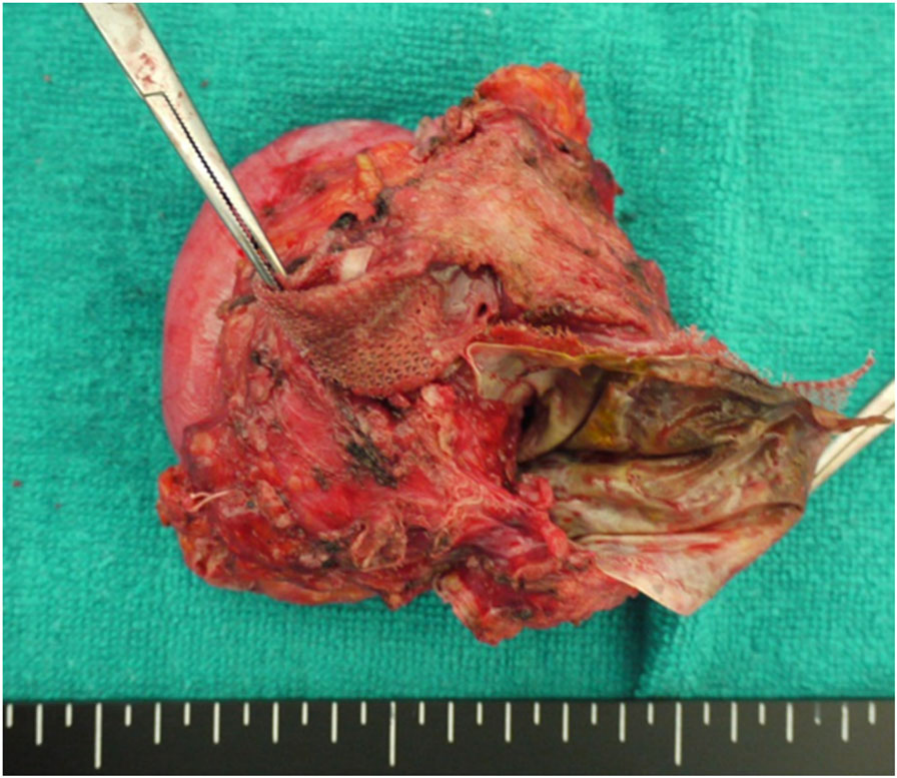
Resected specimen. The mesh was located in the peri-esophageal region and the esophageal lumen

The patient visited our outpatient clinic 1 month after the surgery and then once every 2 months. An upper gastrointestinal endoscopy performed 2 months after the surgery revealed no abnormalities. Abdominal CT was not performed, because the patient was asymptomatic and had no laboratory abnormalities. The patient was seen again at the outpatient clinic of this hospital in March 2012, complaining of dysphagia. Both the WBC count and serum CRP were within normal limits. An upper gastrointestinal series revealed the extrusion of the esophagus in a pocket-like shape with stagnated contrast medium. An upper gastrointestinal endoscopy revealed exposure of the mesh at two sites, 36 and 37 cm distal to the incisors, where ulceration causing an esophageal stricture was also found (Fig. 2). A contrast-enhanced CT showed the extrusion of the mesh into the peri-esophageal region and the esophageal lumen (Fig. 3). When the movability of the mesh using forceps was checked, the mesh was found to be firmly adherent. As no improvement of the stricture was noted despite four sessions of endoscopic esophageal dilatation with an 18-mm balloon, we performed a lower esophagus-proximal gastrectomy and esophagogastrostomy in July 2012 (Fig. 4). We packed the hiatal orifice with a reconstructed gastric tube, leaving no room between the hiatus and the reconstructed gastric tube. A partial hepatectomy was also required because of the obstinate adhesion of part of the mesh to the lateral segment of the liver. Lymphocytic infiltration of the mucosa was the only significant pathologic finding. The patient was discharged home on the 25th postoperative day.

### Discussion

Esophageal hiatal hernias arise as a result of the weakening of diaphragmatic muscular tissues, often because of aging, and can cause reflux esophagitis. The first surgical treatment for reflux esophagitis was reported by the Mayo Clinic in 1911, and many operative procedures have since been described including the Hill repair method [2], the Nissen method [3], the Balsey Mark IV method [4], and the Toupet procedure. With the spread of minimally invasive laparoscopy during the first half of the 1990s and the report on laparoscopic Nissen fundoplication by Dallemagne et al., laparoscopic surgical practice for esophageal hiatal hernia has become increasingly popular [5].

The guidelines for the management of hiatal hernia established by the Society of American Gastrointestinal Endoscopic Surgeons (SAGES) recommend surgical treatment as follows. (1) All symptomatic paraesophageal hiatal hernias should be repaired, particularly those with acute obstructive symptoms or which have undergone volvulus. (2) Routine elective repair of completely asymptomatic paraesophageal hernias may not always be indicated. Considerations for surgery should include the patient’s age and the presence of co-morbidities. (3) Acute gastric volvulus requires the reduction of the stomach with a limited resection, if needed [6]. In the present case, the patient underwent an operation because she had heartburn and respiratory distress as symptoms of a type IV esophageal hiatal hernia.

Simple closure of the hernial orifice by direct suturing has been commonly performed for the repair of the hernia orifice in esophageal hiatal hernia, and cases with recurrence of the hernia have been reported in which repair using a simple closure resulted in the rupture of the orifice plication, while cases with a large hernial orifice have resulted in intrathoracic wrapping or displacement of the esophagogastric junction [7–9]. Repair of a hernial orifice with a mesh has thus been used as a measure to cope with these problems, and Champion and Rock described the efficacy of mesh repair for large hernial orifices exceeding 5 cm [10]. Laparoscopic mesh-augmented hiatoplasty was associated with a lower recurrence rate, compared with a laparoscopic mesh-free hiatoplasty, in a reported by Müller-Stich et al. [11]. In the present case, we performed a laparoscopic Toupet fundoplication and mesh repair for a large esophageal hiatal hernia that could not be closed using a simple laparoscopic closure. The indication for mesh reinforcement in hiatal hernia repair at our hospital was considered for patients with type IV hiatal hernia, large hernial orifice exceeding 5 cm, or fragile hernial orifice.

However, sporadic late complications arising from mesh repair have been reported. Stadlhuber et al. reported 28 cases of postoperative complications after mesh repair for esophageal hiatal hernia. According to their report, the most common chief complaint was dysphagia, followed, in descending order of frequency, by heartburn, chest pain, epigastric pain, and weight loss. Among the reoperated patients, intraesophageal exposure of the mesh occurred in 17 patients, esophageal stricture occurred in 6 patients, and marked fibrosis occurred in 5 patients. No relationship between the development of complications and the type or shape of the mesh used for the repair has been observed [1]. The underlying pathogenetic mechanism is likely as follows: chronic irritation of the esophageal wall and gastric wall caused by the edge of the mesh results in erosions, ulceration, and perforation of the esophageal wall, allowing the passage of the mesh into the gastrointestinal lumen.

We conducted a PubMed search using “hiatal hernia,” “mesh erosion,” and “migration” as key words and retrieved 18 case reports [12–23] of mesh exposure in the esophagus or stomach (Table 1). The mean patient age was 61.6 years (12–84 years), the male to female ratio was 1:1.1, and the mean interval from surgical repair until the onset of complications was about 20 months (7 days–108 months). Dysphagia was the most common chief complaint, encountered in 95% of the cases, followed by weight loss in 30%. Other possible symptoms included epigastric pain, heartburn, regurgitation, and bleeding. Most patients had multiple chief complaints. The mesh was made of PTFE/ePTFE in 14 of the 19 reported cases (74%). All the patients had undergone an endoscopic examination for diagnostic purposes. In the present case, the patient had dysphagia for 13 months after the first operation, with an onset that was earlier than that reported in our review. We had used a composite mesh repair in the primary operation. Champion and Rock [10] reported that polypropylene has a propensity to form adhesions and can erode. In the present case, the PTFE/ePTFE mesh might have been responsible for the mesh migration. We cannot reach a consensus as to which type of mesh should be used for surgical treatment because the relationship between mesh material and mesh erosion has not been previously reported. However, the use of a mesh composed of a soft material might reduce the incidence of mesh migration.

**Table 1:** Reports of mesh-induced esophageal erosion after hiatal hernia repair

Author	Year	Sex	Age	Symptom	Type of mesh	Symptom onset after operation by mesh repair	Place of complication		Removal of mesh	Primary operation	2nd operation	Mesh shape	Fixing method	Diagnostic method
Dutta[12]	2007	M	12	Dysphagia	PTFE	9 years	Esophagus	Laparoscopic and endogastric mesh removal	Yes	Nissenfundplication	Laparotomic Mesh repair	A horseshoe	Non-absorbable	Endoscopy Esophagogram
Griffith [13]	2008	F	84	Dysphagia	ePTFE	7 months	Esophagus	Esophageal stent	NO	Laparoscopic Nissenfundplication with mesh repair	None	-	-	Endoscopy
		F	66	Dysphagia	ePTFE	12 months	Esophagus	Endoscopic removal	Yes	Laparoscopic Nissenfundplication with mesh repair	None	-	-	Endoscopy
		M	69	Dysphagia	-	34 months	Esophagus	Laparoscopic mesh removal	Yes	Nissenfundplication with mesh repair	None	-	-	Endoscopy
Tatum [23]	2008	-	62	Epigastric pain, Dysphagia, Regurgitation	PTFE	3 years	EGJ	Total gastrectomy	Yes	Primary crural closure	Nissenfundplication with mesh repair	-	-	Endoscopy
		-	65	Dysphagia	PTFE	1 months	Esophagus	Surgical mesh removal	Yes	Laparoscopic Nissenfundplication with mesh repair	None	-	-	Esophagogram Manometry
Kepenekci[16]	2009	M	48	Dysphagia,Weight loss, in appetence	Polypropylene	12 months	Esophagus	Distal esophagectomy	Yes	Laparoscopic antireflex surgery with mesh	None	Keyhole collar	-	Endoscopy Esophagogram
Hazebroek[17]	2009	F	80	Dysphagia, Regurgitation	PTFE/ePTFE	4 months	EGJ	Laparotomy mesh removal	Yes	Nissenfundplication	Mesh repair	Heart shape	Titanium helical screws.	Endoscopy
Zugel[18]	2009	M	57	Aortal bleeding	Mono phase	7 days	Abdominal aorta	Laparotomy vascular sutures	No	Laparoscopic mesh repair	None	-	Hernia staplers	Unknown
Holmstrom[15]	2011	M	53	Dysphagia, Wight loss	PTFE/ePTFE	24 months	Stomach	Distal esophagectomy	No	Laparoscopic Nissenfundplication	Nissenfundplication with mesh repair	-	Titanium staplers.	
Arroyo [21]	2011	F	71	Dysphagia, Weight loss	ePTFE	2 years	Esophagus	Gastrostomy	No	Nissenfundplication with mesh repair	None	-	-	Endoscopy Esophagogram
De Moor [19]	2012	M	59	Dysphagia,Fever up	Composite mesh	11 days	Esophagus	Laparotomy mesh removal	No	Laparoscopic mesh repair	None	-	-	Endoscopy Esophagogram CT
		F	58	Epigastric pain, Dysphgea	Composite mesh	16 months	Stomach	Endoscopic removal	Yes	Laparoscopic Nissenfundplication with simple closure	Laparoscopic mesh repair	-	Protack	Endoscopy CT
		M	61	Dysphagia, Weight loss	-	6 months	Stomach	Balloon dilation	No	Laparoscopic antireflex surgery	None	-	-	Endoscopy
Porziella [20]	2012	F	47	Dysphagia,Wight loss	ePTFE	-	Stomach	Endoscopic removal,surgial debridement	Yes	Laparoscopic Nissenfundplication	-	Dog shape	-	Endoscopy Esophagogram
Gandara [14]	2014	F	68	Dysphagia,	PTFE/ePTFE	6 months	EGJ	Endoscopic removal	Yes	Laparoscopic Nissenfundplication with mesh repair	None	-	-	Endoscopy Esophagogram
Liang[22]	2015	M	81	Dysphagia, Regurgitation	-	3 months	Esophagus	Laparoscopic mesh removal	-	Laparoscopic Nissenfundplication with mesh repair	None	-	-	Esophagogram CT Manometry
		F	59	Epigastric pain, Dysphgea	PTFE/ePTFE	6 months	Esophagus	Laparoscopic mesh removal	Yes	Laparoscopic Nissenfundplication with mesh repair	None	-	-	Endoscopy
Our case	2012	F	71	Dysphagia,Heartburn	PTFE/ePTFE	13 months	Esophagus	Esophagectomy	Yes	Laparoscopic Nissenfundplication with mesh repair	None	Round shape	Non-absorbable	Endoscopy Esophagogram CT

*CT* computed tomography, *M* male, *F* female, *PTFP* polytetrafluoroethylene, *ePTFP* expanded polytetrafluoroethylene, *EGJ* esophagogastric junction, - unknown;

Jansen et al. [24] were the first to demonstrate mesh migration into the esophageal wall in a rabbit model. They placed two different types of meshes [polypropylene (PP), Prolene1; polypropylene–polyglecaprone 25 composite (PP-PG), Ultrapro1] on the hiatus as an anterior onlay patch overlapping the hiatal crura at a circular distance of 3 mm from the esophageal wall. The meshes were 2 cm in diameter and were fixed to the diaphragm with four polypropylene (6-0) single stitches. Following this procedure, they found mesh migration into the esophageal wall in six out of seven (86%) animals in the PP group and five out of nine (56%) animals in the PP-PG group. Therefore, it was determined that the distance from the edge of the mesh to the esophageal wall should be more than 3 mm. The larger distance is beneficial for preventing mesh migration, but could also increase the risk of hernia recurrence.

In the present case, the mesh, which was fixed at a distance of 5 mm from the abdominal esophagus, caused esophageal ulceration and eventually became exposed within the esophagus, resulting in dysphagia. The movement of the mesh in the present case might have resulted from chronic irritation exacerbated by peristaltic movements, since the mesh had been fixed close to the esophagus. Therefore, it was suggested that the distance from the edge of the mesh to the esophageal wall should be more than 10 mm to reduce the risk of mesh-induced injuries clinically and practically.

As for the type of mesh selected, friction between the mesh, which was made of a hard material, and the esophageal wall was likely to have exacerbated the irritation of the esophageal wall, resulting in the development of the complication. In cases with postoperative symptoms such as dysphagia and heartburn associated with elevated inflammatory markers on blood biochemistry tests, an upper gastrointestinal endoscopy and CT examination should be considered to rule out possible recurrences or complications caused by the mesh placement, leading to the early discovery of this complication.

While some meta-analyses and systemic reviews have reported on mesh-related complications after laparoscopic repair of giant hiatal hernias, there are no reports comparing the mesh-related complications between patients undergoing laparoscopic repair and those undergoing open repair of giant hiatal hernias [25–27]. We believe that adoption of the laparoscopic approach may not be a major cause of this mesh-related complication.

Various reports have described treatments for complications arising from mesh-induced injuries. About 82% of patients with recurrences underwent a reoperation, according to the report by Stadlhuber et al. [1]. The most frequently performed surgical procedure was an esophagectomy, but other procedures included mesh removal by total gastrectomy, partial gastrectomy, or laparotomy. Non-operative procedures that were reported included endoscopic removal and stent placement. Our literature review showed that reoperation was necessary in 68% of cases, and the mesh was removed endoscopically in 21% of the cases. Stent placement and balloon dilation were performed in 2 of the 19 reported cases (10%). Endoscopic balloon dilatation is not a radical treatment for esophageal stenosis caused by a mesh. Rather than endoscopic treatment, mesh excision should be performed to obtain a good outcome. In the present case, we attempted to remove the mesh during an upper gastrointestinal endoscopy using balloon dilation. Our attempt failed, however, because of the presence of firmly adherent tissue and fibrous tissue incorporation. Surgery should be considered when a cure cannot be achieved endoscopically. The methods used to treat complications, including surgery, should be decided in accordance with the disease condition, and reoperation has been undertaken in many cases; therefore, the selection of the mesh, including consideration of its material composition, at the time of the initial operation and the position and distance from the esophageal hiatus at which the mesh is fixed are of clinical importance.

## Conclusion

Currently, a wide variety of mesh types are available, and guidelines for the appropriate use of meshes are being examined; therefore, the further accumulation of cases is necessary. Simple plication without the use of a mesh should be considered as the method of first choice for preventing mesh-related complications. Nevertheless, for cases with a large esophageal hiatal hernia, in which the use of a mesh cannot be avoided, it is important to select a soft, durable mesh and to secure it firmly in place at a position distant from the gastrointestinal wall.

## Abbreviations

CT: Computed tomography
EGJ: Esophagogastric junction
ePTFP: Expanded polytetrafluoroethylene
F: Female
M: Male
PTFP: Polytetrafluoroethylene
